# Protein Structure-Guided Hidden Markov Models (HMMs) as A Powerful Method in the Detection of Ancestral Endogenous Viral Elements

**DOI:** 10.3390/v11040320

**Published:** 2019-04-02

**Authors:** Heleri Kirsip, Aare Abroi

**Affiliations:** 1 Department of Bioinformatics, University of Tartu, Tartu, 51010, Riia 23, Estonia; 2 Institute of Technology, University of Tartu, Tartu, 50411, Nooruse 1, Estonia

**Keywords:** endogenous viral elements, bioinformatics, horizontal gene transfer, virus-to-host gene transfer, HMM, tobacco mosaic virus, *Drosophila*, capsid protein

## Abstract

It has been believed for a long time that the transfer and fixation of genetic material from RNA viruses to eukaryote genomes is very unlikely. However, during the last decade, there have been several cases in which “virus-to-host” gene transfer from various viral families into various eukaryotic phyla have been described. These transfers have been identified by sequence similarity, which may disappear very quickly, especially in the case of RNA viruses. However, compared to sequences, protein structure is known to be more conserved. Applying protein structure-guided protein domain-specific Hidden Markov Models, we detected homologues of the Virgaviridae capsid protein in Schizophora flies. Further data analysis supported “virus-to-host” transfer into Schizophora ancestors as a single transfer event. This transfer was not identifiable by BLAST or by other methods we applied. Our data show that structure-guided Hidden Markov Models should be used to detect ancestral virus-to-host transfers.

## 1. Introduction

Viruses are one of the most abundant and prevalent biological entities on Earth and thus are an important and integral part of the biosphere (for viral importance in environments, see [1,2,3,4]). The importance of viruses for humans and the biosphere is illustrated by the fact that, in our bodies, there are 10 times more bacteria than human cells (~10^13^ cells), and the number of viruses is even higher than the number of bacteria by an order of magnitude [5]. The same prevalence also applies to viruses in other biotopes; for example, there are approximately 10^8^ viruses in one litre of water [6] and approximately 10^6^–10^7^ viruses per m^3^ in the atmosphere [7]. In most biotopes, the number of viruses exceeds the number of prokaryotes by an order of magnitude [4]. The overall virus–host interaction is an important aspect driving viral evolution and the role of viruses in evolution. These roles are not restricted to the host–parasite arms race. It has been known for a long time that bacteriophages mediate transduction in bacteria. It is also known that retroviruses integrate into their hosts’ genomes and thus can affect the genomic organisation of the vertebrate’s cells [8]. However, Retroviridae is one viral family among approximately one hundred viral families that infect eukaryotes, and vertebrates constitute only a small fraction of eukaryotes. What is less known is that other, non-retroviral viruses are able to do the same, i.e., integrate viral genes into the chromosomes of infected cells, both in vertebrates and in other eukaryotic cells, of course not in such direct way as retroviruses. [9,10]. The viral genome elements that have integrated into eukaryotic genomes and have become fixed are called endogenous viral elements (EVEs) [9]. It has been shown that both cytoplasm- and nucleus-replicating non-retroviral viruses with different replication strategies ((+)ssRNA, (−)ssRNA, dsRNA, ssDNA and dsDNA viruses) can integrate into animal [11,12], plant [13] and fungal [14] genomes (for a thorough review, see [15]). Thus, the integration of viral genes into eukaryotic genomes is quite widespread. In contrast, the function and expression of retroviral EVEs have only been thoroughly researched in model organisms [16], and for non-retroviral EVEs, additional analyses of EVE function and expression have been performed only for human and squirrel endogenous bornavirus-like nucleoprotein (EBLN) elements [17,18,19] and tobacco plant geminivirus-related DNA sequences (GRDs) [9,20]. The exact pathway by which viral genes integrate and become fixed in eukaryotic organisms is not clear. However, to be effectively spread over the population, EVEs must also benefit the organism (although spreading via genetic drift, founder effects and population bottlenecks cannot be excluded).

The main strategy for detecting EVEs in organisms was formalised by Kondo et al. [21]. The detection protocol for EVEs consists of three main stages: (a) EVE detection with bioinformatics tools, mostly with BLAST searches in different databases; (b) EVE detection and confirmation in experiments, mostly with PCR and DNA sequencing to confirm integration; and (c) EVE phylogeny construction combined with viral sequences to identify EVEs closest relatives and to evaluate the integration time.

Following this protocol, scientists have discovered a large amount of viral-like sequences in different eukaryotic genomes. However, there is a problem with detecting distant homologues, which was noted by Brenner et al.; BLAST does not work well with sequences under 30% of identity [22]. Park et al. [23] showed that Intermediate Sequence Search (ISS) method, Hidden Markov Models (HMMs) and PSI-BLAST work considerably more effectively than pairwise sequence comparison methods (e.g., BLAST), especially when the sequences have changed greatly over time. Profile-HMMs are probabilistic models that can be used to describe the patterns shared by sets of protein/domain sequences (HMMs are generated from constructed multiple sequence alignments (MSAs), and the generated model covers both the diversity and conservation within the input MSA). It is possible to search for a sequence that is the most similar to the model, which is especially important when dealing with viral sequences because the virus mutation speed is much higher, especially in RNA viruses (approximately 10^−2^ to 10^−7^ substitutions per position per year compared to about 10^−9^ substitutions per position per year in mammals [24,25,26]), and confident pairwise similarity between homologous sequences may disappear. Thus, profile-HMMs should detect distant homologous sequences more efficiently.

Advantages of other methods in comparison with BLAST in the case of viruses have been shown in practise. Kuchibhatla et al. started from the assumptions above and used sequence-profile comparisons (such as HMMER3) and profile–profile comparisons (HHpred) in addition to BLAST analyses [27]. They showed that sequences that were previously classified as “ORFans” (more accurately “taxonomically restricted to only one taxon”) according to BLAST analyses, had distant homologues in various viruses and that this approach could also be applied to organisms. Thus, considering the fast evolution of viruses, profile-based algorithms that can detect distant homologues should be more suitable for detecting (distant) homologues of viral proteins [28,29].

It is also known that protein structure is more conserved than sequence [30,31]. Therefore, the protein structure is also important to consider when detecting distant homologous sequences. When dealing with the identification of similarities between evolutionarily distant, but homologous sequences, taking protein structure-based information into account may improve the results. Challis and Schmidler showed that, when including structural information, the phylogenetic inference for distant relationships improves [32]. Additionally, Herman et al. showed that structure contains more information that can be obtained from sequences; hence, it vastly reduces the alignment and phylogenetic tree topology uncertainty [33]. This finding is especially important in the case of viruses, which lose sequence similarity within a relatively short time, especially compared to organisms. Thus, structural information should be included when studying deep evolutionary relationships.

In this study, we tested whether a complex method, such as profile-HMM together with protein structure-guided information, is a suitable and effective first step in EVE detection and whether it should be implemented in the EVE-detection protocol. To complete this aim, a comparison between BLAST and profile-HMM search results was made with the objective of finding whether profile-HMMs could detect more distant EVEs and how many more EVEs are identified when implementing a structure-guided profile-HMM search. This work shows that using profile-HMMs with additional structure-guided information enables the detection of more distant homologous sequences; in this case, endogenous *Tobamovirus* (family Virgaviridae; +ssRNA viruses) coat protein-like (eTCPL) elements in different fly genomes that have not been detected previously by using pairwise sequence comparison method BLAST.

## 2. Materials and Methods

### 2.1. EVE Detection Using BLAST Analysis

**Figure 1:**
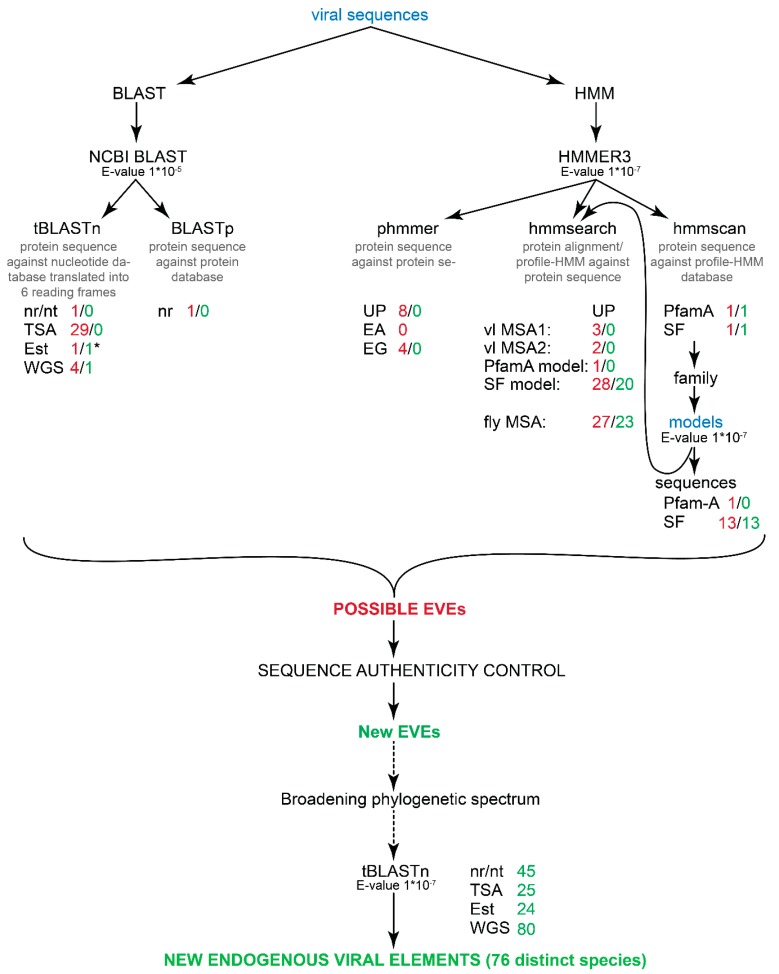
Workflow of the identification of new EVEs. The first step is the search against eukaryotic sequences from different databases (the Swiss-Prot database results are left out because this database gave no hits) using viral sequences as a query. All the search inputs (sequences and models) are indicated in blue script. All the hits are then examined to determine false positives and mis-annotations. All the numbers after the databases (written in red) indicate the distinct hit IDs that were collected from the databases. The second number (written in green) indicates (after all the noted sequence authenticity controls) how many possible EVEs were identified from the databases. The “*” denotes one recombinant sequence that was found in the analyses. “vl MSA1” denotes *Tobamovirus*-specific MSA, “vl MSA2” denotes *Tobamovirus*-*Hordeivirus*-*Pecluvirus*-*Tobravirus* specific MSA and “fly MSA” denotes an MSA that was constructed using 13 *Drosophila* fly sequences from the SUPERFAMILY database. Abbreviations: UniProtKB, UP; Ensembl All, EA; Ensembl Genomes, EG; SUPERFAMILY, SF. Note that “nr” database in BLASTp search include also UniProtKB. The sequence authenticity control workflow is described in detail in Figure 2.

To detect eukaryotic endogenous *Tobamovirus* coat protein-like (eTCPL) elements, the BLAST method was used (Figure 1). Different *Tobamovirus* (family Virgaviridae, +ssRNA viruses), *Hordeivirus* (family Virgaviridae, +ssRNA viruses), *Tobravirus* (family Virgaviridae, +ssRNA viruses) and *Pecluvirus* (family Virgaviridae, +ssRNA viruses) coat proteins (CPs) were used as queries (the sequences used are shown in Table S1) in NCBI BLASTp and tBLASTn (protein sequence against the nucleotide database translated into 6 reading frames) (BLAST+ 2.2.30+, updated 6.10.2014; using the NCBI BLAST server http://blast.ncbi.nlm.nih.gov/ [34]) searches in eukaryotes (NCBI Taxonomy ID 2759). The search was performed against nucleotide collection (nt/nr), Transcriptome Shotgun Assembly (TSA), expressed sequence tag (Est) and whole-genome shotgun contig (WGS) databases using default parameters with an additional implemented *E*-value threshold of 1 × 10^−5^ (May 2017) (results in Table S2). additionally, BLASTp (protein sequence against protein database) was run using the same query sequences and search parameters (including an *E*-value threshold of 1 × 10^−5^) against a non-redundant protein database (“nr”, which also includes most of the UniProtKB sequences). Searches and databases used are visualised in Figure 1.

### 2.2. EVE Detection Using the Profile-HMM Method

As an alternative method, searches were also performed with the HMMER3 package (Figure 1) (version 1.8; using the web server http://www.ebi.ac.uk/Tools/hmmer/ [35]). The searches were performed using the same *Tobamovirus*, *Hordeivirus*, *Tobravirus* and *Pecluvirus* CP sequences as described above for BLAST analyses with taxonomic restriction to Eukaryotes (NCBI Taxonomy ID 2759) using the default parameters with an additional *E*-value threshold of 1 × 10^−7^.

In the “phmmer” search type (protein sequence against protein sequence database), the databases used were UniProtKB (version v.2017_05), Swiss-Prot (version v.2017_05), Ensemble All (version v.88) and Ensemble Genomes (version v.35) because no other databases were available during the analyses (May 2017) (results in Table S3).

In “hmmscan” (protein sequence against the profile-HMM database), viral sequences were used to search against protein families in the Pfam-A [36] and SUPERFAMILY [37] databases (April 2017). Both resources contain protein domain-specific HMM profiles and assign these profiles to available protein sequences in different databases. The “hmmscan” search results give the protein domain-specific HMM profile ID. Further, it is also possible to analyse these HMM profiles and the sequences that are assigned to the profile. Eukaryotic sequences assigned to these profiles can be analysed as possible eTCPLs. The *E*-value threshold for belonging to the respective domain family was set to 1 × 10^−7^ (results in Table S4). 

For the “hmmsearch” search (protein alignment/profile-HMM against protein sequence database), the *Tobamovirus* CP MSA and the *Tobamovirus*-*Pecluvirus*-*Tobravirus*-*Hordeivirus* CP MSA were constructed using the MUSCLE algorithm (with default parameters) within MEGA (version 7.0.20; [38]). The search was performed in the UniProtKB and Swiss-Prot databases (April 2017) (results in Table S5). Next, “hmmsearch” was performed using previous “hmmscan” results (predefined protein family models from the Pfam-A/SUPERFAMILY databases) as queries (May 2017) (results in Table S6).

### 2.3. Testing for False Positive Hits with Alternative Methods

**Figure 2:**
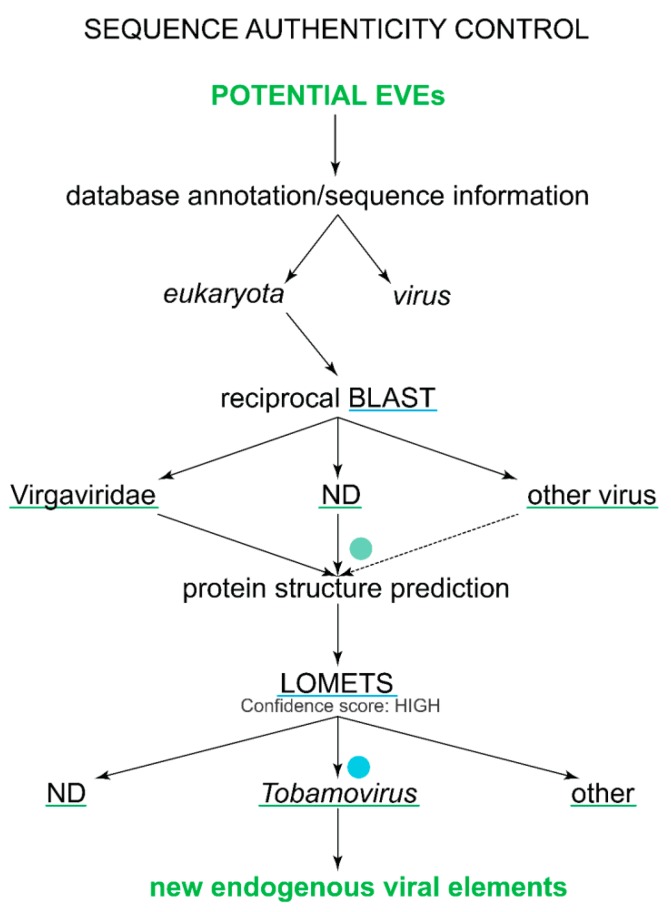
A detailed sequence authenticity control workflow. Each step describes what kind of results can be obtained from each step and what results can be used in further analyses. The programs used in the analyses are underlined with blue line and the results of the analyses are underlined with green line. The green dot shows that we cannot distinguish between viruses and EVEs when only RNA-seq data are available. Similarly, the blue dot shows that, even when protein structure prediction may indicate that the sequence is homologous to a certain virus, this sequence may have a close relative whose structure is not yet solved. However, the protein structure prediction still indicates that the potential EVE sequence most likely belongs to the respective structural superfamily (in terms of the SCOP).

As stated by Pearson, “When a scientifically unexpected alignment appears to be statistically significant, investigators should consider alternate strategies for estimating statistical significance” [39]; we applied alternative methods to confirm the homology of high scoring hits (Figure 2). First, to test for annotation artefacts, database annotations of sequence’s scaffold/contig/chromosome (their origin), overall length, sequence location and surrounding area were scanned. Data confirmation was needed to exclude the possibility of sample contamination that could have occurred during cloning or sequence assembly and/or a misannotation of the protein itself (for example, nucleic acid is isolated from tissues infected with a virus, but all sequences are still annotated as host sequences).

Second, to exclude false positive hits (sequences that are more similar to other sequences or domains), a reciprocal sequence similarity search was performed with default parameters using tBLASTn for protein sequences (against the NCBI nr/nt database) and BLASTn (nucleotide sequence against the nucleotide database) for nucleotide sequences (against the NCBI nr/nt database). When the reciprocal search gives the most significant hit to primary query sequences or their close relatives (in viruses in the same viral genus or family), it is noted as such in all of the tables and in other cases, the best hit is described; additionally, “no hits” are marked with “−”. Other viral hits that were not closely related to Virgaviridae viruses were classified as false positives for eTCPL but will remain possible positive hits for other EVEs and will be analysed in the future. Additionally, a “hmmscan” (protein sequence against the profile-HMM database) search was performed on the HMMER webpage to test whether the sequence belongs to another protein domain family with much higher confidence.

Third, to determine possible false positive results with alternative methods, protein structure prediction was performed with the LOMETS [40] meta-server for all remaining positive hits from both the BLAST and HMMER3 analyses. Additionally, this step was important for the positive hits (both eukaryotic and viral hits) obtained from the SUPERFAMILY and Pfam-A databases to confirm that profile-HMM models have not falsely assigned sequences to the models. The LOMETS meta-server simultaneously uses many different algorithms to predict possible protein structures from sequences using the protein threading method. If the sequence is a real homologue, the server should recognise the tobamoviral coat protein or its structural relatives in SCOP, i.e., the protein domains with “SCOP concise classification string” (SCCS) (also named “SCOP superfamily identifier”) staring with “a.24.5” as a template for high-confidence models. Additionally, the confidence score, the alignment length and the template coverage should be high, and at least one algorithm not based on HMMs should give a high confidence score. In addition, the *Z*-score was considered, for which a higher score is considered to be better than a lower score. All possible eTCPL hits that were not classified as false positives or non-determined (ND) were used in the phylogenetic analyses.

In general, all the sequences that reciprocal BLAST classified as similar to tobamoviruses or other Virgaviridae but only had RNA data available were classified as ND because we cannot distinguish between actual viral sequences and new EVEs.

### 2.4. Phylogenetic Analysis for eTCPL

For the phylogenetic analysis, a joint list of viral coat protein sequences and eukaryotic EVE sequences were used. Viral sequences were taken from the NCBI full genomes database, belonging to tobamoviruses, pecluviruses, hordeiviruses, tobraviruses and goraviruses (Table S1).

The eukaryotic sequences used in this study are from two datasets. The first dataset consists of eukaryotic sequences from the SUPERFAMILY database that have been assigned to eTCPL elements (Table S7). Sequences that were included in the phylogeny were those that remained after the false positives were excluded. For these sequences, only the eTCPL region that was assigned to the SUPERFAMILY HMM model was used. The second dataset (search results) consists of the HMMER analysis results (Table S5) that were obtained when using Virgaviridae viral MSAs as queries. One additional sequence (*Lasioglossum albipes*; NCBI accession ID: ANOB01025386.1), which was the one positive hit from the tBLASTn analyses using the viral sequence as a query, was added to the second dataset. All phylogenetic analyses were performed using the protein domain part of the sequences (*Drosophila melanogaster*, FlyBase gene ID FBgn0029799, UniProt ID Q9W483, protein region 122–259). To determine the domain part of the sequences (where the domain part was not previously designated), the two datasets were merged and aligned within MEGA using ClustalW and were edited using the first eukaryotic dataset as a baseline to cut out the regions that were outside of the eTCPL protein domain. Additionally, Jalview [41] was used to remove identical sequences (remove redundancy threshold of 100%).

All the results were additionally filtered to ensure data quality because most of the partial domains in proteins are alignment and/or annotation artefacts [42]. Protein sequences shorter than 130 AA were removed and not used in the MSA. All sequences were aligned using the Mafft alignment algorithm (version 7) with the default parameters [43]. To select the best model for phylogeny construction, the ProtTest server was used [44]. The best model was selected using the Akaike information criterion framework: LG + I + G + F. The phylogenetic tree was constructed using the MEGA program package with maximum likelihood methods using LG with frequencies (+F) model along with the gamma distribution with invariant sites (G + I). The number of discrete gamma categories was 5. Gaps and missing data were dealt with via partial deletion using a site coverage cut-off of 95%. The initial tree was performed automatically (using the NJ/BioNJ method). For the phylogeny test, the bootstrap method (500 replications) was used.

### 2.5. Additional BLAST Analysis to Broaden the Search to Databases that Are not Accessible in HMMER3

For the final step in detecting EVEs, an additional BLAST analysis was performed to identify potential EVEs not yet annotated as proteins (Figure 1). This analysis was done because the HMMER3 web server has few directly connected databases, none of which are nucleotide databases. Hence, performing an additional BLAST analysis enables us to broaden the phylogenomic coverage to incomplete genomes and/or nonannotated proteins. Eukaryotic sequences (Table S8), obtained through protein domain databases (SUPERFAMILY), were used as queries to search for eukaryotes (NCBI Taxonomy ID 2759) using tBLASTn with the same parameters described previously and with *E*-value 1 × 10^−7^.

### 2.6. eTCPL Synteny in Complete Fly Genomes

To determine the possible integration events in further detail, completely sequenced and annotated genomes that harbour potential eTCPLs were explored in more detail. From all the results, only *Drosophila* species were fully sequenced; thus, they were used in the synteny analysis. All the work was performed using the FlyBase database [45] and its genome browser (June 2017). eTCPL genes in corresponding fly genomes were determined, and the gene regions surrounding it on both sides were analysed. Additionally, the copy number of the eTCPLs, the localisation of the surrounding genes and the mobile element existing near the eTCPL site were included in the analysis.

## 3. Results

### 3.1. Searching for EVEs Using “Sequence versus Sequence” Search Algorithms (BLAST and “Phmmer”)

First, to identify potential homologues of *Tobamovirus* coat proteins (tobamo-CPs) from eukaryotic genomes, a BLAST analysis [34], as a standard protocol for detecting EVEs, was used. In general, the viral protein sequences were used as queries in BLAST analyses (a list of viral sequences used can be found in Table S1), but the analyses gave no informative *Tobamovirus*-related eukaryotic results (Figure 1 and Table S2). In BLASTp (protein vs. protein search), one result (UniProt ID: P93362_TOBAC) had a significant E-value and bit-score; however, closer examination of the annotation showed that its viral sequences have been annotated as a part of the *Nicotiana tabacum* plant genome.

We extended our search to tBLASTn (protein sequence as a query against nucleotide database translated into 6 reading frames) to find EVEs not yet annotated or not coding a protein. Using tobamo-CP as a query in tBLASTn against the nr/nt database, no true positives were found. However, against WGS (Whole Genome Sequence) database, four significant hits were obtained (Figure 1 and Table S2). In reciprocal BLAST analyses, one sequence was highly similar to Polydnaviridae, and two of the sequences seemed to be relatives of the tobamo-CP. However, since the contig length of the DNA was similar or shorter than the known *Tobamovirus* genome length, these sequences were classified as ND (non-determined because of a lack of information to confidently classify this sequence as part of the eukaryotic genome). One of these, *Lasioglossum albipes* (bee) sequence ANOB01025384.1 (NCBI accession ID) in the WGS database, however, did have a DNA scaffold over 40 Mbp (40,000 bp) in length. Protein structure prediction of the region that harbours the potential EVE indicated that the tobamoviral coat protein was the best match. However, the database annotations also state that DNA was extracted from the whole body; hence, it is currently not possible to determine whether the scaffold is part of the actual bee genome or is from some other organism found in the bee’s holobiont.

We also performed a similar tBLASTn search against the TSA (Transcription Shotgun Assembly) and Est (Expressed Sequenced Tags) databases. No true positive hits were obtained because, while sequencing the whole-body RNA, no evidence was given that the RNA was transcribed from the eukaryotic genome (Figure 1 and Table S2).

To determine whether using a more complex method (while still using the protein sequence as a query) can give us more information than a pairwise comparison method, additional analyses were performed. Hidden Markov Models have been shown to identify distant homologous sequences more successfully than BLAST. HMMER3 [35] was used for this purpose because it has been shown to be more sensitive while not losing computational speed. When using the same *Tobamovirus* coat protein sequences as queries in HMMER3 “phmmer” search against the protein sequence databases, no new significant results were found (Table S3), but this result may be due to the small number of databases that can be searched against (UniProt, SwissProt and Ensemble). None of the 18 primary hits from this analysis met the other criteria described in the Materials and Methods Section.

Thus, sequence versus sequence searches gave one possible true hit (*Lasioglossum albipes*; NCBI accession ID: ANOB01025384.1). Despite expanding the search queries to include *Hordeivirus* (family Virgaviridae; +ssRNA viruses), *Pecluvirus* (family Virgaviridae; +ssRNA viruses) and *Tobravirus* (family Virgaviridae; +ssRNA viruses) capsid proteins (sequences listed in Table S1), no additional EVEs were found.

### 3.2. Searching for EVEs Using “MSA versus Sequence” search Algorithms (“hmmsearch”)

As noted above, it has been known for a long time that profile-based methods work better than sequence-based methods, especially in distant homologous sequence detection. We used *Tobamovirus* and *Tobamovirus*-*Hordeivirus*-*Pecluvirus*-*Tobravirus* capsid proteins (sequences listed in Table S1) to generate MSAs and the corresponding profiles, and then we searched the sequence databases using these profiles. All these analyses gave hits (Table S5) that had been previously classified as false positives, whether by viral contamination or by belonging to other protein domains. Hence, the “MSA versus sequence” search did not result in any new EVEs (Figure 1).

### 3.3. Identification of EVEs Using Profile-HMMs from the Pfam-A Database

Additionally, when searching single sequences against databases that use protein domain profile-HMMs (e.g., Pfam-A, TIGRFAM, and SUPERFAMILY), HMMER3 “hmmscan” gave highly reliable results when using the tobamo-CP as a query (Table S4). The Pfam-A database gave the result of protein family “Virus coat protein (TMV like)” (Pfam-A code PF00721.20). This Pfam-A family consists of sequences of *Tobamovirus*, *Hordeivirus*, *Pecluvirus*, *Tobravirus* (Virgaviridae, +ssRNA virus), *Furovirus* (Virgaviridae, +ssRNA virus), *Pomovirus* (Virgaviridae, +ssRNA virus) and *Benyvirus* (Benyviridae, +ssRNA virus) coat proteins. However, this Pfam-A family does not contain any eukaryotic sequences (neither in Pfam-A version 28 nor in versions 29, 30 and 31), except for one *Nicotiana tabacum* protein (UniProt accession ID: P93362_TOBAC) that has previously identified as a viral sequence. Pfam-A version 31 is based on UniProt release 2016_10 and does not include sequences deposited after October 2016. To overcome this limitation, we used Pfam-A’s own profile-HMM model as a search query against the UniProt database (“hmmsearch”) on the HMMER webpage (https://www.ebi.ac.uk/Tools/hmmer/), which uses a monthly updated version of the UniProt. However, no new results were found (Table S6).

### 3.4. Searching for EVEs Using Structure Guided Profile-HMMs from the SUPERFAMILY Database

SUPERFAMILY is the other profile-HMM database, where the protein domain family is presented as a profile-HMM [37]. It identified “TMV-like viral coat proteins” (SUPERFAMILY accession ID: 47195 or SF_47195) as a positive hit (Table S4). SUPERFAMILY is a resource of genomic assignment of protein structural domains. This database constructs profile-HMMs for protein structural domains at the SCOP [46] superfamily level (throughout this article, we used term “superfamily” as it is defined in SCOP) and then uses completely sequenced and annotated genomes to identify distant homologous sequences that belong to the same structural superfamily. According to the SCOP authors, a superfamily level groups protein domains with a common ancestor.

According to the SCOP classification, the superfamily “TMV-like viral coat protein” (SCOP concise classification string “sccs” a.24.5) is composed of four *Tobamovirus* coat protein structures (PDB ID: 1EI7, 1VTM, 1RMV, 1CGM). Obviously, the *Tobamovirus* capsid sequences (22 genomes) are assigned to HMM models of this superfamily. Additionally, the capsid sequences of tobraviruses (three genomes), hordeiviruses (one genome) and pecluviruses (two genomes) are assigned to the “TMV-like viral coat protein” superfamily. Additionally, three sequences from Bymoviruses (Potyviridae, +ssRNA virus), each from different genomes, gave highly significant hits. However, these sequences are not capsid proteins but are from a RNA2-encoded polyprotein (P2). Recall that SUPERFAMILY resources use non-redundant, complete viral genomes from NCBI as a source for viral sequences.

In addition to viruses, thirteen *Drosophila* fly species are also assigned to “TMV-like viral coat protein” superfamily (Table S7). All thirteen *Drosophila* species were assigned with very high confidence (an *E*-value less than 1 × 10^−33^) and with an almost full alignment length. Thus, these sequences are most likely homologous to tobamo-CPs. Although this database uses completely sequenced and annotated genomes and the profile-HMMs are trained to be highly specific, false positives classifications should not be overlooked; hence, protein structure prediction was performed to confirm the SUPERFAMILY database results. Additionally, when using HMM models of the SF_47195 SCOP classification from the SUPERFAMILY database as queries in the “hmmsearch” on the HMMER webpage with up-to-date databases, several hits were found (Table S6). Most of these species belonged to different *Drosophila* species, and others belonged to different fly species from the Schizophora section (*Bactrocera* species, *Ceratitis capitata*, *Lucilia cuprina*, *Musca domestica*, *Stomoxys calcitrans* and *Glossina* species). Similar results were also observed when using the SUPERFAMILY *Drosophila* sequence (protein domain part)-based MSA as the “hmmsearch” query (Table S5).

### 3.5. Confirming SUPERFAMILY Hits as True Homologues to TMV-CP

To exclude false positives, the LOMETS metaserver [40] was chosen for protein structure prediction (Figure 2). This program uses protein threading, which attempts to fit the given sequence to previously identified protein structures, aligns each amino acid accordingly, and then it evaluates how well the target fits the template. The results of the LOMETS metaserver for these *Drosophila* sequences show that the best modelling templates, according to several different prediction algorithms (including those that do not use HMMs), belong to tobamo-CP structures. This result confirms that *Drosophila* fly sequences are highly likely to have structures very similar to tobamo-CPs and thus have a high likelihood of being real homologues.

When looking at the surrounding region of the eTCPL in *Drosophila* (and in other true positive hits for SUPERFAMILY HMMs) in more detail (i.e., when analysing contig length and the presence of other viral domains), no other indication of a misannotated viral origin was found. Thus, the *Drosophila* eTCPL is coded by the real part of the genome and not by viral sequences accidently annotated as a part of the genome. The eTCPL is found in several *Drosophila* species, which significantly reduces the probability of sequencing artefacts. In conclusion, the SUPERFAMILY HMMs found several true positives among Diptera.

### 3.6. Phylogenetic Analysis of Virgaviridae CP and Diptera eTCPLs

The above analysis shows that *Drosophila* and other flies contain the eTCPL domain. This result by itself does not address transfer direction. To evaluate the phylogenetic relationship of eTCPLs and exogenous viruses, we performed a comprehensive phylogenetic analysis using CP sequences of corresponding Virgaviridae viruses (tobamoviruses, tobraviruses, pecluviruses, hordeiviruses and goraviruses) (Table S1); and the integrated eTCPLs in eukaryotes (only high-confidence eTCPLs were used).

**Figure 3:**
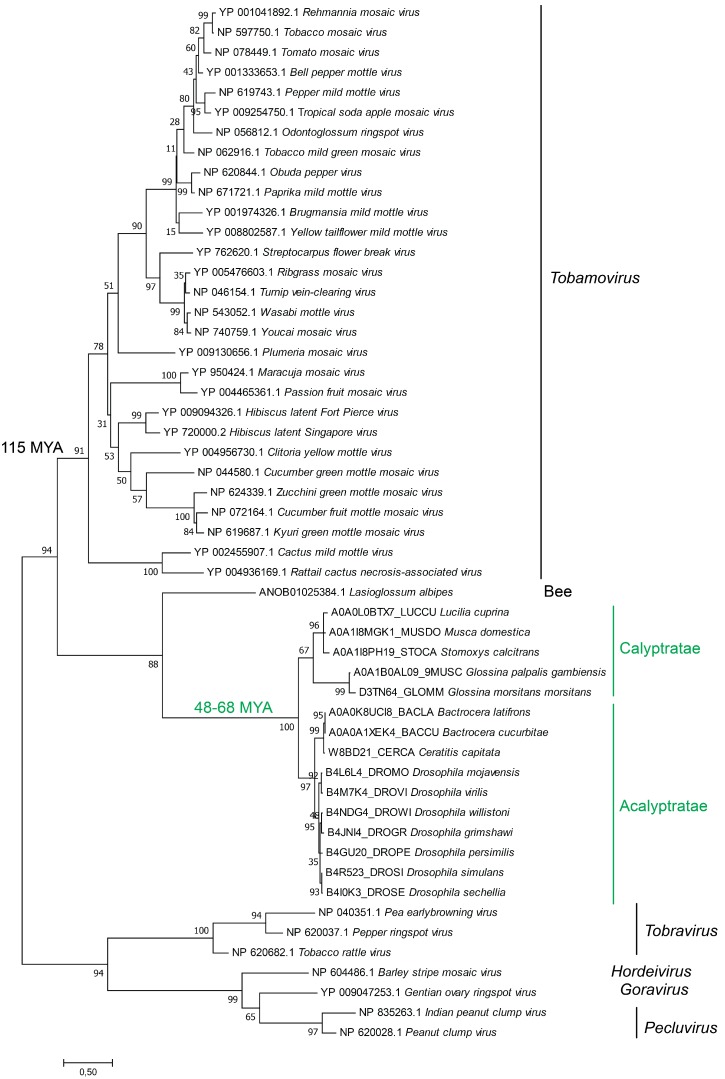
Phylogeny of eTCPLs and the corresponding viral sequences. The tree was constructed using the maximum likelihood method (LG + I + G + F model) using the MEGA program package [48]. All viral genera are monophyletically grouped according to their phylogeny. All the eukaryotic sequences are also grouped, and the pattern follows known fly phylogeny. The Acalyptratae-Calyptratae divergence from other flies [49,50,51,52] and *Tobamovirus* divergence from other Virgaviridae [53,54] have been noted.

As shown in the phylogenetic tree in Figure 3, all viral genera (tobamoviruses, hordeiviruses, pecluviruses and tobraviruses) have been clearly separated according to their known phylogeny. The overall distribution of viral genera is very similar to the known divergence of these viruses, with hordeiviruses and pecluviruses being very closely related, as determined based on both replication proteins and capsid protein phylogenies of Virgaviridae [47]. These data show that the phylogenetic analysis is biologically significant and meaningful.

All flies are distributed to two main groups based on the two subsections of Schizophora flies: Acalyptratae and Calyptratae. All the eTCPLs from different flies cluster together with very high confidence and form a sister clade with extant viruses. The phylogenetic pattern inside the eTCPL clade is consistent with the divergence of flies in the Calyptratae and Acalyptratae subsections and resembles the phylogeny of the flies itself [55].

It has been shown that the Acalyptratae-Calyptratae split occurred approximately 63–68 MYA according to three independent studies [49,50,51]. A more focused and through study showed that the split occurred approximately 48 MYA [52]. On the other hand, the ancestral split of *Tobamovirus* from other Virgaviridae has been shown to have occurred approximately 115 MYA [53,54]. Overall, when the phylogenetic tree is supplemented with available divergence dates, it provides strong support for the virus-to-host transfer.

### 3.7. eTCPL Synteny in Completely Sequenced Fly Genomes

**Figure 4:**
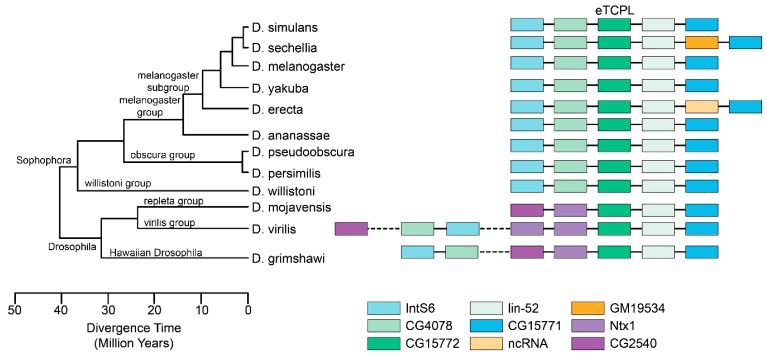
eTCPL gene synteny comparisons on fully sequenced genomes from *Drosophila* flies. The eTCPL is noted in dark green and was taken as an observation centre. *Drosophila melanogaster*, the most well-researched fly, was taken as a baseline and two to three genes surrounding the eTCPL were also considered. All data were collected from the FlyBase database [45], including the tree picture. In some flies whose genes surrounding the eTCPL were different, previously noted genes (IntS6 and CH4078) were also located for this analysis.

In this work, eTCPLs have been found in many different fly genomes. Of these, only a few genomes have been fully sequenced, assembled and deposited into the FlyBase database [45]. All of them are from *Drosophila* species (Table S7). Using their assembled genomes, the eTCPLs and the surrounding region were analysed. All the eTCPLs are present in a single copy, and the protein domain itself is coded by a single exon (in *Drosophila willistoni* and *Drosophila ananassae*, the respective genes have two exons, but the eTCPL domain is coded by a single exon). The genes surrounding *Drosophila melanogaster* eTCPL are IntS6, CG4078, CG15771 and lin-52 (the eTCPL gene overlaps with the last gene but not in the coding sequence). The same trend is apparent in all of the *Drosophila* species (Figure 4). However, some changes have occurred in either *Drosophila* or the *Sophophora* subgenus. For *Drosophila virilis* and *Drosophila grimshawi*, the genes IntS6 and CG4078 are located further away and are located in different scaffolds for *Drosophila mojavensis*. Additionally, *Drosophila sechellia* acquired a new gene (GM19534) that has no known orthologues. Henceforth, a possible viral gene could have integrated in this locus before the *Drosophila* fly split.

### 3.8. EVE Detection from Incompletely Sequenced Genomes

True positive eukaryotic hits of the SF_47195 from the SUPERFAMILY database (which uses the complete genomes and UniProt) indicated flies are hosts to eTCPLs. The next step in the analysis was an additional BLAST analysis to evaluate their phylogenomic distribution in uncomplete genomes, whose proteins may not yet be annotated.

In our approach, HMMs can recognise deeper evolutionary relationships, and BLAST will give a better phylogenomic coverage since the HMM search was limited to a few protein databases and BLAST has a much wider possible database usage range and allows searching against nucleotide databases (including subsets that do not have annotated translations). Using the fly eTCPLs as queries, 76 different species (including 13 species from SUPERFAMILY) were identified as containing true positive eTCPLs in their genome (Table S8). The results mostly consist of both Acalyptratae and Calyptratae species (65 species belonging to the Schizophora section) covering both of their phylogenies as best as possible with the data available.

Most of the species (in the Schizophora group) also have the same genes surrounding the eTCPLs as in *Drosophila melanogaster* (genes IntS6, CG15771 and CG4078 located on the same contig as the eTCPL) with the exception of a few species (data not shown), most likely because those species are not sequenced as fully as others. This also indicates that eTCPLs’ synteny shown for *Drosophilidae* (Figure 4) is also extended to Calyptratae and thus for all Schizophora.

We can use protein structure-guided profile-HMM analyses with an additional tBLASTn search to cover the un-annotated coding sequences. With this approach, it is possible to detect eTCPL sequences in a large number of eukaryotic genomes, which was not possible when using only a BLAST search.

## 4. Discussion

During the last decade, many EVEs have been detected in eukaryotes. According to the published literature, the number of viral families that have donated EVEs to eukaryotes is at least 34 for identified nucleic acid transfers, including at least 21 viral families where the coding potential of integrated viral sequences have remained (data collected from the literature by H. Kirsip). The recipient eukaryotic organisms have been fungi, plants, vertebrates, arthropods and others. Even when using sequence-based EVE search strategies, the impact of non-retroviral viruses in cellular genomes/proteomes is (and has been) significant. Currently applied methods do not take into account that viruses have very fast evolutionary rates, and thus, sequence similarity may not be detectable using pairwise sequence comparison methods.

The data presented in this study show that, as expected from the literature, there exist methods that are more sensitive than BLAST to detect “virus-to-host” transfers of genetic material. It should be noted that the same method could also be applied to the “host-to-virus” approach. According to the literature, HMM-based searches should be more sensitive than BLAST, and structure-guided HMMs should be even more sensitive than sequence-based HMM searches in the detection of remote homologues. In our case, BLAST found one possible homologue in eukaryotes for *Tobamovirus* capsid proteins. Additionally, using different HMMER3 search capabilities (“phmmer” and the viral MSA-based “hmmsearch”, both based on HMMs) did not give any new reliable results. At the same time, structure-guided HMMs from SUPERFAMILY found distant homologues of tobamoviral proteins in eukaryotes. Our data show that, as a “proof of principle”, structure-guided HMMs can be used to detect V2H transfers that are not detectable by other methods.

In modern high throughput times, the annotations of the data in different databases vary, particularly from the point of view that relevant aspects of annotations vary for different researchers (and for their scientific questions). In our case, the authenticity of sequences (their origin) must be carefully tested because very often the taxonomic annotations of the origin of a nucleic acid correspond to major species in holobionts and not to their real origin. This phenomenon has also been used by scientists in a positive way, extracting, for example, the viruses (real viruses, not EVEs) infecting insects from the data with taxonomic annotation to insects [56]. If the sequence data are not linked to publications (or if the methods of how the probe was isolated and prepared have not been properly described), it is very hard to evaluate the real origin of a sequenced nucleic acid. Some types of data, by definition, do not allow us to distinguish between viruses and EVEs (for example, RNA-seq data). Curated datasets such as complete proteomes or complete genomes seem to have fewer un-authentic sequences compared to WGS. However, using WGS and similar databases are very useful for acquiring taxonomic coverage that is as wide as possible. Of course, every method may have false positive hits; therefore, we analysed all the search hits with multiple alternative methods to obtain more confidence results. Unfortunately, these methods are also not as “high throughput”.

Structure-guided HMMs help to detect remote homology; however, detecting homology is not enough to classify the sequence as an EVE. In addition to vertical inheritance from primordial worlds, there are two processes that may lead to homologous proteins in viruses and organisms: “virus-to-host” transfer and “host-to-virus” transfer. To detect EVEs, the transfer direction of homologues found in viruses and cells must be determined. Determination of the transfer direction for more ancestral transfers is not as trivial as in the case of more recent events. In the latest case, it is possible to use the highly confident outgroup sequences to root the phylogenetic tree and polarised it on time. At the same time, when dealing with more older events, determining a good outgroup is a challenge. Studying the evolutionary history of a single viral protein (or a protein domain) selection of outgroups is complicated by rampant horizontal gene transfer between viruses. Even such an unlikely event as gene transfer between ssDNA viruses and RNA viruses has been reported [10,57,58]. Known divergence times of cellular and viral taxa would also help to polarise the phylogenetic tree. Unfortunately, very few virus group divergence times have been determined. Therefore, to determine the transfer direction, different independent approaches should be used.

To determine the transfer direction of proteins under study, we combined several approaches. First, we tested the synteny of eTCPLs in well-annotated species in the FlyBase database. The eTCPL and its surrounding area in fully sequenced genomes were analysed. The data in Figure 4 show the synteny of the eTCPL in these species. In different *Drosophila* fly genomes, the eTCPL is in the same locus, is coded by a single exon, and the surrounding genes are similar. The exception is between *Drosophila* and the *Sophophora* subgenus, where some gene region reorganisation has occurred in one of them. Respective species (where the eTCPL has the same location according to FlyBase) diverged approximately 48 MYA (the known divergence time for *Drosophila* species) [52], and at least for these species, the eTCPL shows a clear monophyletic origin with a single transfer event according to the proposed phylogeny of the *Drosophila*/*Sophophora* common ancestor. We also reconstitute the phylogenetic tree of viral tobamo-CP and cellular eTCPL sequences together. This tree (and several other trees with different alignment and tree building algorithms [59]) shows very strong bootstrap confidence intervals for viral genera as well as for the Calyptratae/Acalyptratae split (from an alignment with a length of ~130 AA), indicating the biological significance of the tree. Seeing as both Schizophora subsections (Acalyptratae and Calyptratae) have eTCPL in their genomes and the eTCPL phylogeny is consistent with known fly phylogeny [55], it can be concluded that the integration of viral elements occurred before these two groups diverged. One group of flies, Aschiza, diverged before the Acalyptratae/Calyptratae divergence and are closest relatives of Schizophora. In this group of flies, there are three organisms with fully sequenced genomes (according to the NCBI Genomes database): *Megaselia abdita*, *Megaselia scalaris* and *Eristalis dimidiata*. None of the organisms could be identified as carrying the eTCPL gene; however, the neighbouring genes of eTCPL were detected using BLAST [59]. Therefore, it could be concluded that the main integration of the tobamo-CP gene was into the ancestor of the Schizophora flies. Supplementing the phylogenomic tree with known divergence times of tobamoviruses, the Calyptratae/Acalyptratae split, and synteny analysis, tobamoviruses are much older than eTCPLs in Schizophora. Therefore, taking all this information into account, the most likely single transfer event took place between 115 and 48 MYA with a direction from viruses to insects.

The presence of plant virus genes in insect genomes may seem very unlikely, but we must take into account that insects are common plant virus vectors and otherwise have very intimate contact with plants. According to that, the described transfer is no longer unexpected. Additionally, single V2H transfer events in one organism must be somehow beneficial for hosts to be fixed and to spread in populations and maintained over millions of years. The detected EVE seems to be biologically active, as the respective mRNA has been observed in several fly species (Table S8). In *Drosophila melanogaster*, the expression is observed in different developmental stages and different tissues (our in silico analysis of preexisting data deposited in different databanks, data not shown). In addition, the peptide corresponding to amino acids 229–246 in UniProt Q9W483 (a product of gene CD15772, i.e., The *D. melanogaster* eTCPL) have been observed in adult *Drosophila melanogaster* heads according to a search in the PeptideAtlas database (www.peptideatlas.org).

However, more detailed experiments need to be performed to determine the full function of the eTCPLs in flies.

## 5. Conclusions

This work showed that fast evolving viral protein homologues can be identified in eukaryotic genomes using structure-guided HMM searches, even when the integration event itself is very ancient.

## Supplementary Materials

The following are available online at https://www.mdpi.com/1999-4915/11/4/320/s1, Supplementary File 1; containing Tables S1–S8.
